# Dopamine Receptors Modulate Cytotoxicity of Natural Killer Cells via cAMP-PKA-CREB Signaling Pathway

**DOI:** 10.1371/journal.pone.0065860

**Published:** 2013-06-14

**Authors:** Wei Zhao, Yan Huang, Zhan Liu, Bei-Bei Cao, Yu-Ping Peng, Yi-Hua Qiu

**Affiliations:** Department of Physiology, School of Medicine, Nantong University, Nantong, Jiangsu Province, China; University of Medicine and Dentistry of New Jersey, United States of America

## Abstract

Dopamine (DA), a neurotransmitter in the nervous system, has been shown to modulate immune function. We have previously reported that five subtypes of DA receptors, including D1R, D2R, D3R, D4R and D5R, are expressed in T lymphocytes and they are involved in regulation of T cells. However, roles of these DA receptor subtypes and their coupled signal-transduction pathway in modulation of natural killer (NK) cells still remain to be clarified. The spleen of mice was harvested and NK cells were isolated and purified by negative selection using magnetic activated cell sorting. After NK cells were incubated with various drugs for 4 h, flow cytometry measured cytotoxicity of NK cells against YAC-1 lymphoma cells. NK cells expressed the five subtypes of DA receptors at mRNA and protein levels. Activation of D1-like receptors (including D1R and D5R) with agonist SKF38393 enhanced NK cell cytotoxicity, but activation of D2-like receptors (including D2R, D3R and D4R) with agonist quinpirole attenuated NK cells. Simultaneously, SKF38393 elevated D1R and D5R expression, cAMP content, and phosphorylated cAMP-response element-binding (CREB) level in NK cells, while quinpirole reduced D3R and D4R expression, cAMP content, and phosphorylated CREB level in NK cells. These effects of SKF38393 were blocked by SCH23390, an antagonist of D1-like receptors, and quinpirole effects were abolished by haloperidol, an antagonist of D2-like receptors. In support these results, H89, an inhibitor of phosphokinase A (PKA), prevented the SKF38393-dependent enhancement of NK cells and forskolin, an activator of adenylyl cyclase (AC), counteracted the quinpirole-dependent suppression of NK cells. These findings show that DA receptor subtypes are involved in modulation of NK cells and suggest that D1-like receptors facilitate NK cells by stimulating D1R/D5R-cAMP-PKA-CREB signaling pathway and D2-like receptors suppress NK cells by inhibiting D3R/D4R-cAMP-PKA-CREB signaling pathway. The results may provide more targets of therapeutic strategy for neuroimmune diseases.

## Introduction

Dopamine (DA), a neurotransmitter in the nervous system, has been reported to modulate immune function besides its conventional regulation of behavior, movement, endocrine, cardiovascular, renal and gastrointestinal functions. We have previously shown that T lymphocytes, one population of adaptive immune cells, are modulated by DA via its receptors [1]. Other reports have also presented an extensive regulation of T cells by DA. For example, DA increases interleukin (IL)-10 production and decreases IL-12 production [2]; DA inhibits the release of IL-2, IL-4 and interferon (IFN)-γ from T cells by activation of D2 and D3 receptors [3], and induces a down regulation of IFN-γ-producing cells [4]. Unlike T lymphocytes, natural killer (NK) cells are one population of innate immune cells, and their function is characterized by the defense against and the kill of viruses and malignancy that parasitize cells. The characteristics of NK cells killing viral or tumor cells are termed cytotoxicity, which is important for protection of the body against viral infection and malignant invasion. Administration of DA significantly enhances the ability of NK cells to kill tumor cells in vitro [5]. APO-SUS rats with a hyperdopaminergic phenotype show a decreased NK cell activity [6], and DA transporter (DAT)−/− mice also display a reduced NK cell activity [7]. These findings represent a regulation of NK cells by DA and also suggest that the regulatory effect of DA on NK cells is manifold. The different regulatory effects on lymphocytes can be caused by activation of different DA receptor subtypes on these cells.

DA receptors are seven-transmembrane G protein-coupled receptors. At the present, five subtypes of DA receptors, including D1R and D5R, classified into D1-like receptors, and D2R, D3R and D4R, classified into D2-like receptors, have been identified [8], [9]. Human and murine leukocytes express the five DA receptor subtypes [10]–[13]. Among the leukocyte subpopulations, T lymphocytes and monocytes have low, neutrophils and eosinophils have moderate, and B lymphocytes and NK cells have high and more consistent expression of D2-D5 receptors [11]. Stimulation of D1/5 receptors not only inhibits cytotoxic function of CD8^+^ T cells [14] but also impairs differentiation and function of regulatory T cells (Tregs) [15], [16]. On the contrary, D2 receptor activation promotes production of IL-10, which is involved in the polarization toward Tregs [17]. These results support that DA receptor subtypes induce various regulatory effects on T cells. However, regarding NK cells that have higher expression of D2-D5 receptors, functional significance of the DA receptor subtypes is poorly clear.

It has been known that downstream signaling of DA receptors is related to cAMP. In general, D1-like receptors link to stimulatory G protein (G_s_), which increases intracellular cAMP and in turn cAMP induces phosphokinase A (PKA) activation, while D2-like receptors couple to inhibitory G protein (G_i_), which decreases intracellular cAMP [18]. We have previously found that via reduction of cAMP and cAMP-response element-binding (CREB), a transcriptional factor that mediates cAMP-induced gene expression via binding to cAMP-response element in gene promoter region, D2-like receptors exert a regulatory effect on T lymphocytes, suggesting that cAMP-CREB signaling pathway is involved in modulation of T lymphocytes by DA [1]. Other reports also present that via the mechanism of D1-like receptor-mediated stimulation of intracellular cAMP, DA inhibits significantly proliferation and cytotoxicity of CD4^+^ and CD8^+^ T cells in vitro [14], [19]. However, whether the DA receptor-coupled signaling pathway is also implicated in regulation of NK cells still remains to be clarified. Thus, the investigation exploring that roles of DA receptor subtypes and their coupled signaling pathways in modulation of NK cells may help better understanding of DA immunomodulating mechanisms and provide more targets for therapeutic strategy of neuroimmune diseases.

## Materials and Methods

### Ethics Statement

The animal work done in this study followed the National Institute of Health Guide for the Care and Use of Laboratory Animals and was approved by the Institutional Animal Care and Use Committee of Nantong University.

### Separation and Purification of NK Cells

Negative selection using magnetic activated cell sorting was employed to enrich NK cells from splenocytes by NK Cell Isolation kit (BD Biosciences, USA). Briefly, spleens of ICR mice were harvested by celiotomy, and single cell suspensions were obtained by gently squeezing the spleens. The Biotinylated Mouse NK Cell Enrichment Cocktail stained erythrocytes and most leukocytes except NK cells. After washing away excess antibody, BD IMag Streptavidin Particles Plus-DM were added to the cell suspension and bound to the cells bearing the biotinylated antibodies. The tube containing the labeled cell suspension was placed within the magnetic field of the BD IMagnet. Negative selection was then performed to enrich the unlabeled NK cells. The enriched NK cells were resuspended in the complete culture medium, RPMI 1640 medium containing 10% heat-inactivated calf serum (Gibco, USA). The cultures were incubated in an incubator (ESPEC BNA-311, Japan) with 5% CO_2_ at 37°C for 4 h.

### Drug Treatment

SKF38393 and quinpirole, the agonists of D1-like and D2-like receptors, respectively, was added to NK cell suspensions of 4×10^6^ cells/ml at a concentration of 10^−8^ or 10^−7^ M. The antagonists of D1-like and D2-like receptors, SCH23390 and haloperidol, respectively, were added to the cultures 30 min anterior to the agonist application at a concentration of 10^−8^, 10^−7^ or 10^−6^ M. Alternatively, H89, an inhibitor of PKA, or forskolin, an activator of adenylyl cyclase (AC), was applied to the NK cell cultures 30 min anterior to the agonist application at a concentration of 10^−8^ or 10^−7^ M. The cultures were incubated in the incubator with 5% CO_2_ at 37°C for 4 h. These drugs were all from Sigma, USA.

### Flow Cytometric Assay for Assessment of NK Cell Cytotoxicity

Cytotoxicity of NK cells against the tumor target cells, YAC-1 cell line that is a Moloney leukemia virus-induced mouse lymphoma with noted sensitivity to NK cells, was measured by flow cytometric assay as described by [20]. NK cells were separated and purified as mentioned above. YAC-1 cell line, a commercial source from Shanghai Institute of Biochemistry and Cell Biology, Chinese Academy of Sciences, was maintained in continuous suspension culture in the complete culture medium at a concentration of about 8×10^5^ cells/ml at 37°C in a humidified 5% CO_2_ incubator. All cultures were split 24 h before use to ensure that the YAC-1 cells were in an exponential growth phase during the assays. The experiments employed two fluorescent stains, calcein acetoxymethyl (CAM, from Fluka, USA) and ethidium homodimer-1 (EH-1, from Fluka, USA). CAM, which readily enters cells and is converted by intracellular esterase into calcein that produces an intense green signal (530 nm), was firstly added to the YAC-1 cells at a concentration of 100 nM, which was incubated for 15 min at 37°C in 5% CO_2_, protected from light. After labeling, the YAC-1 cells were washed twice, counted and adjusted to 4×10^5^ cells/ml. NK cells of 0.5 ml (4×10^6^ cells/ml) was added to the CAM-labeled YAC-1 cells (0.5 ml), and the mixtures were incubated at 37°C in the humidified 5% CO_2_ incubator for 2 h. EH-1, which binds to DNA of dead cells and emits red fluorescence (617 nm), was then applied to the mixtures of CAM-stained YAC-1 cells and NK cells at a concentration of 200 nM for 15 min at room temperature. Four groups of the cells were thus identified. Intact NK cells were non-fluorescent, dead NK cells emitted red fluorescence, living YAC-1 cells exhibited green fluorescence, and dead YAC-1 cells were characterized by double (green-red) fluorescence. Flow cytometry was performed with a FACS Calibur (BD Biosciences, USA) equipped with an argon laser operating at 488 nm. Two parameter dot plots were obtained with cellQuest software (BD Biosciences, USA). The NK cell cytotoxicity was expressed as percentage of specifically dead YAC-1 cells relative to total YAC-1 cells.

### Real-time Quantitative Polymerase Chain Reaction (PCR)

Expression of DA subtype receptors, D1R, D2R, D3R, D4R and D5R, at mRNA levels in NK cells was examined by quantitative real-time PCR. Total RNA in NK cells was extracted with Trizol reagent (Invitrogen, Carlsbad, CA, USA) according to the manufacturer’s instructions. Potentially contaminating residual genomic DNA was eliminated with RNAse-free DNAse (Promega, Madison, WI, USA). After the RNA content was determined by spectrophotometric analysis at 260 nm, 2 µg of total RNA was used for cDNA synthesis with murine myelomonocytic lymphoma virus reverse transcriptase (Promega, Madison, WI, USA). The single-stranded cDNA was then amplified by real-time quantitative PCR for evaluation of relative expression levels of five genes of the interest. The PCR reaction was performed in a Rotor-Gene 3000 Real-Time Cycler (Corbett Research, Australia). The binding of the fluorescence dye SYBR Green I (Molecular Probe, Eugene, OR, USA) to double-stranded DNA was measured. Each 20 µl of reaction mixture contained 1 µl of cDNA synthesized as above, 2 µl PCR buffer, 3.0 mM MgCl_2_, 0.2 mM of each dNTP, 0.2 µM of each pair of oligonucleotide primers (Table 1), and 1 U Taq DNA polymerase. The reaction procedures were as follows: an initial step at 95°C for 5 min, 40 cycles of 94°C for 30 s, 60°C for 30 s and 72°C for 30 s. The data was collected using the instrument’s software (Rotor-Gene software, version 6.0) and relative quantification was performed using the comparative threshold (CT) method after determining the CT values for reference (β-actin) and target genes (D1R, D2R, D3R, D4R and D5R) in each sample sets according to the 2^−ΔΔCt^ method [21], as described by the manufacturer (User Bulletin). Changes in mRNA expression levels were calculated after normalization to β-actin. As calibrator sample we used cDNA from control cells without any treatment. The program calculates the ΔCts and the ΔΔCt with the formulas below:
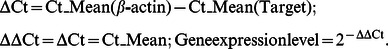



**Table 1:** Sequences of PCR primers.

Gene	Sense primer	Antisense primer	Product size (bp)	Accession No.
D1R	5′-TGTGACACGA GGTTGAGC-3′	5′-GGTGGTCTG GCAGTTCTT-3′	177	NM_010076
D2R	5′-CCATTGTCTG GGTCCTGT-3′	5′-TGCCCTTGAG TGGTGTCT-3′	258	NM_010077
D3R	5′-CTACGCCCTG TCCTACTGT-3′	5′-CCACCTGTCA CCTCCAAG-3′	189	NM_007877
D4R	5′-GTGTTGGACG CCTTTCTTCG-3′	5′-GGGTTGAGGG CACTGTTGA-3	120	NM_007878
D5R	5′-CTGCGAGCAT CCATCAAG-3	5′-CACAAGGGAA GCCAGTCC-3′	160	NM_013503

### Western Blot Analysis for DA Receptor Expression and CREB Phosphorylation Level

Nuclear lysates were extracted from NK cells. Protein concentration was estimated by a modified Bradford assay, and 20 µg of protein was boiled for 5 min in 2×loading buffer. The protein was separated by sodium dodecyl sulfate-polyacrylamide gel electrophoresis and transferred to a polyvinylidine difluoride membrane (Pall, USA) using a semi-dry transfer apparatus. After blocking nonspecific bindings with 5% (w/v) nonfat dry milk, the membranes were probed with antibodies specific for D1R, D2R, D3R, D4R and D5R (all from Santa Cruz, USA) or for phosphorylated CREB and total CREB (both from Cell Signaling Technology, USA) at room temperature for 2 h or at 4°C overnight. They were incubated with a fluorescently-conjugated secondary antibody for 1 h at room temperature and visualized by Odyssey laser scanning system (LI-COR Inc, USA). The molecular weight and relative quantity of the protein bands were determined by an image analysis system (Odyssey 2.1, LI-COR Inc, USA).

### Radioimmunoassay for cAMP Content

After incubation for 4 h, 1×10^6^ NK cells/ml were collected and subjected to centrifugation. The cell pellet was treated with 1 ml of 50 mM acetic acid (pH 4.75), which was stored at −20°C until analysis. The cells, broken by repeated freezing and thawing, were centrifuged at 3,000×g for 15 min at 4°C. The supernatants were measured for cAMP levels by radioimmunoassay using a commercially available kit (Beijing North Institute of Biological Technology, Beijing, China). Briefly, portions of samples (100 µl/sample) were acetylated and then incubated with 100 µl ^125^I-cAMP (20,000 cpm/100 µl) and 100 µl of anti-cAMP antibody at 4°C for 24 h. Rabbit serum (100 µl) and goat anti-rabbit IgG (100 µl) were added to the mixture, which was incubated for 12 h at 4°C and then centrifuged at 3,000×g at 4°C for 20 min. Radioactivity of the deposits was measured by use of a multi-well gamma counter (Shanghai Rihuan Instrument Company, Shanghai, China).

### Statistical Analysis

Data were expressed as means ± standard deviation (M ± SD). Statistical analyses were performed with the Statistics Package for Social Science (SPSS, 12.0). The data were subjected to the one-way analysis of variance (ANOVA), followed by Student-Newman-Keul’s test to compare the data of all groups relative to each other. Differences were considered statistically significant at p<0.05.

## Results

### Expression of DA Subtype Receptors in NK Cells

**Figure 1:**
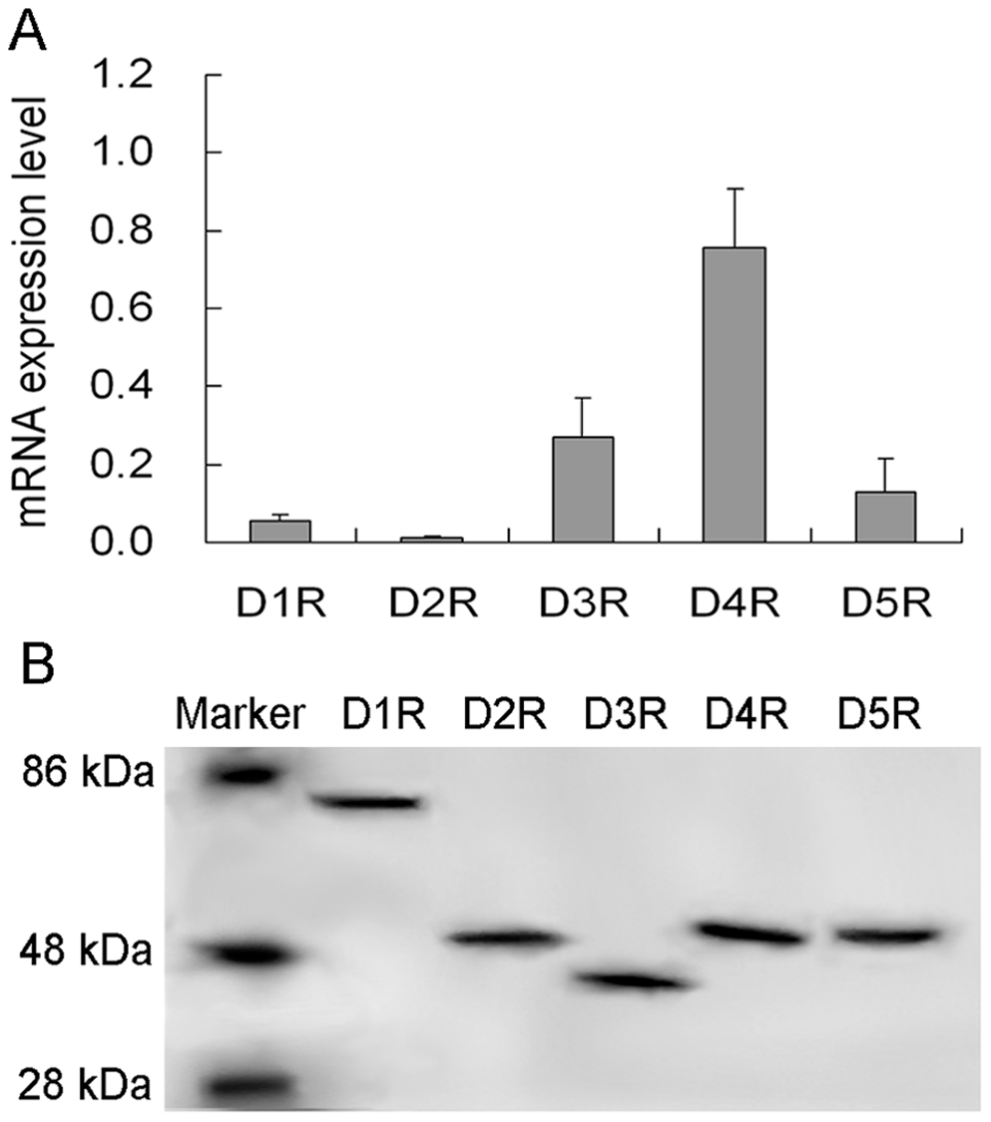
Expression of DA subtype receptors in NK cells. NK cells from mouse spleen were purified and detected for expression of D1R, D2R, D3R, D4R and D5R at mRNA and protein levels by using real time-PCR and Western blot, respectively. (A) The data was normalized to β-actin and represent M ± SD of three repeated experiments. (B) The electrophoretic bands show expression of the five DA receptor subtypes in NK cells and the molecular weights are 74, 51, 44, 53 and 53 kDa for D1R, D2R, D3R, D4R and D5R, respectively, which are consistent with those predicted.

Data from real-time PCR exhibited that purified NK cells from the spleen expressed five subtypes of DA receptors, including D1R and D5R (D1-like receptors), as well as D2R, D3R and D4R (D2-like receptors) (Fig. 1). Correspondingly, these five subtypes of DA receptor proteins were also expressed by NK cells (Fig. 1). Their molecular weights were consistent with those predicted, i.e., 74, 51, 44, 53, and 53 kDa for D1R, D2R, D3R, D4R, and D5R, respectively.

### D1-like Receptor Agonist SKF38393 Enhances NK Cell Cytotoxicity and this Effect is Blocked by D1-like Receptor Antagonist SCH23390

**Figure 2:**
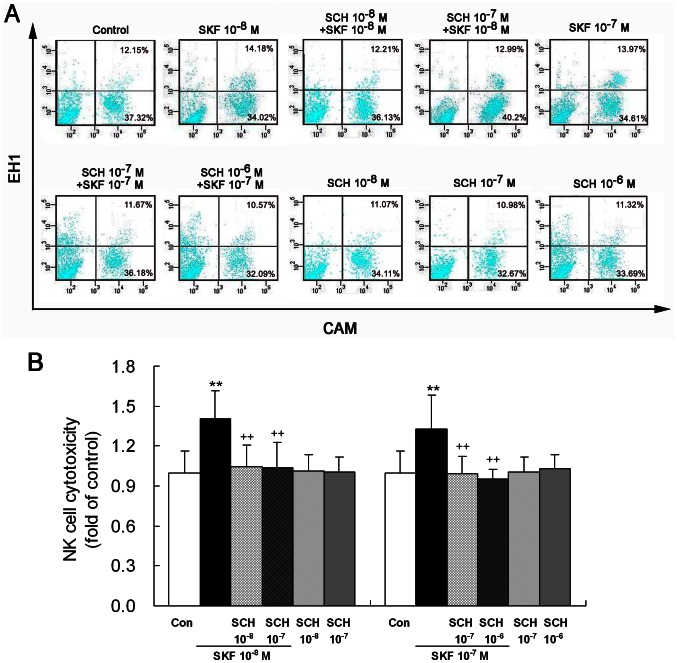
Activation of D1-like receptors enhances NK cell cytotoxicity. NK cells were treated with D1-like receptor agonist SKF38393 (10^−8^ or 10^−7^ M) or co-treated with the antagonist SCH23390 (10^−8^, 10^−7^ or 10^−6^ M) and SKF38393 for 4 h. Flow cytometric analysis was used to determine cytotoxicity of NK cells against YAC-1 lymphoma cells. (A) A representative diagram showing fluorescent dot images for various treatments. In each treatment, the four plots denote percentage of different cells. The upper left plot reflects dead NK cells labeled by EH-1; the low left plot represents intact NK cells labeled by neither CAM nor EH-1; the upper right plot shows dead YAC-1 cells stained by both CAM and EH-1; and the low right plot indicates living YAC-1 cells stained by CAM. The NK cell cytotoxicity was expressed as percentage of specifically dead YAC-1 cells relative to total YAC-1 cells. (B) A statistical graph of eight independent experiments as were performed in (A). **p<0.01, vs. control (Con); ^++^p<0.01, vs. SKF38393 (SKF). SCH: SCH23390.

To show functional significance of DA subtype receptors expressed in NK cells, we examined effect of D1-like receptor agonist SKF38393 on cytotoxicity of NK cells against the target cells, YAC-1 lymphoma cells. The exposure to SKF38393 (10^−8^ or 10^−7^ M) significantly enhanced cytotoxicity of NK cells (Fig. 2).

To confirm that the SKF38393 effect was caused by activating D1-like receptors, we applied the joint treatment with D1-like receptor antagonist SCH23390 (10^−8^, 10^−7^ or 10^−6^ M) and the agonist SKF38393 (10^−8^ or 10^−7^ M). The combined treatment significantly reduced NK cell cytotoxicity compared with the treatment with SKF38393 alone, with a return of the cytotoxicity of NK cells to control level (Fig. 2). This result indicated that SCH23390 blocked SKF38393-dependent enhancement of NK cell cytotoxicity. The exposure to SCH23390 alone did not significantly alter the cytotoxicity of NK cells relative to control (Fig. 2). These data demonstrate that the enhanced NK cell function by the agonist SKF38393 is associated with D1-like receptor activation.

### SKF38393 Elevates D1R and D5R Expression, cAMP Content and CREB Phosphorylation in NK Cells, and these Effects are Blocked by SCH23390

**Figure 3:**
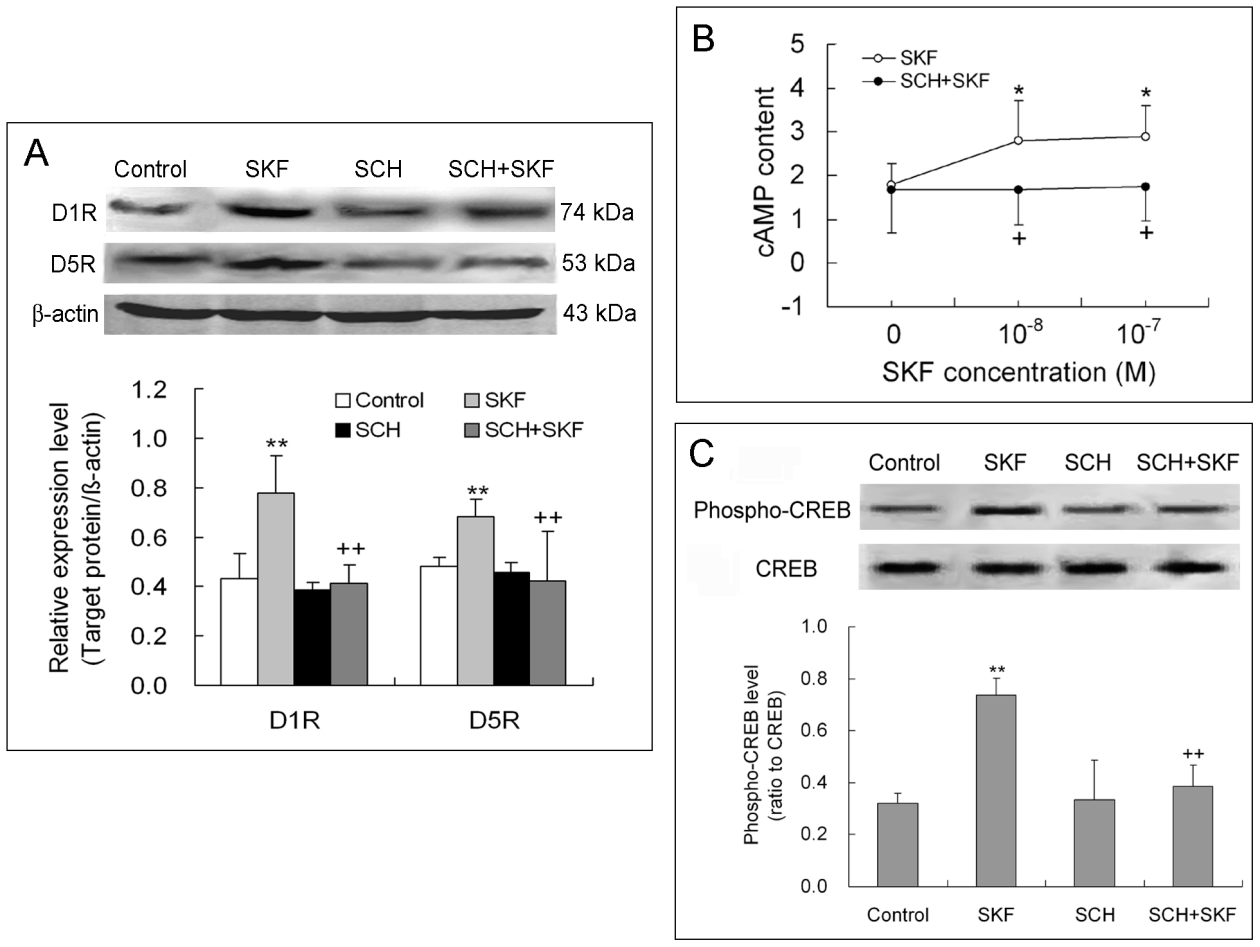
Activation of D1-like receptors elevate D1R and D5R expression, cAMP content, and CREB phosphorylation level in NK cells. NK cells were treated with D1-like receptor agonist SKF38393 (10^−8^ or 10^−7^ M) or with antagonist SCH23390 (10^−7^ M) plus SKF38393 for 4 h. Expression of D1R and D5R and level of CREB phosphorylation were detected by Western blot, and cAMP content in NK cells was measured by radioimmunoassay. (A) Representive electrophoretic bands and statistic chart of three independent experiments for D1R and D5R expression in NK cells. (B) The data showing cAMP content in NK cells were obtained from eight separate samples for each treatment. (C) Representive electrophoretic bands and statistic graph of three respective experiments for phosphorylated CREB level in NK cells. *p<0.05, **p<0.01, vs. control; ^+^p<0.05, ^++^p<0.01, vs. SKF (SKF38393). SCH: SCH23390.

To reveal signaling pathway mediating the D1-like receptor-dependent enhancement of NK cell cytotoxicity, we detected changes in expression of D1-like receptors (including D1R and D5R), cAMP content and CREB phosphorylation level in NK cells after the cells were treated with D1-like receptor agonist SKF38393 (10^−8^ or 10^−7 ^M). Compared with control cells lacking treatment, the NK cells exposed to SKF38393 displayed an up-regulated expression of D1R and D5R proteins, an increased cAMP content, and a raised CREB phosphorylation level (Fig. 3). These results indicate that SKF38393 stimulates D1R/D5R-cAMP-CREB signaling pathway in NK cells.

In addition, co-exposure to D1-like receptor antagonist SCH23390 (10^−7^ M) and agonist SKF38393 (10^−8^ or 10^−7^ M) prevented the SKF38393-induced increase in D1R and D5R expression, in cAMP content and in CREB phosphorylation (Fig. 3). The exposure to antagonist SCH23390 alone did not remarkably affect D1R/D5R expression, cAMP content or CREB phosphorylation in NK cells (Fig. 3). The blocking effect of SCH23390 on SKF38393-stimulated D1R/D5R-cAMP-CREB signaling confirms that this pathway is associated with D1-like receptor activation.

### PKA Inhibitor H89 Suppresses NK Cell Cytotoxicity and Prevents the SKF38393-Dependent Enhancement of NK Cells

**Figure 4:**
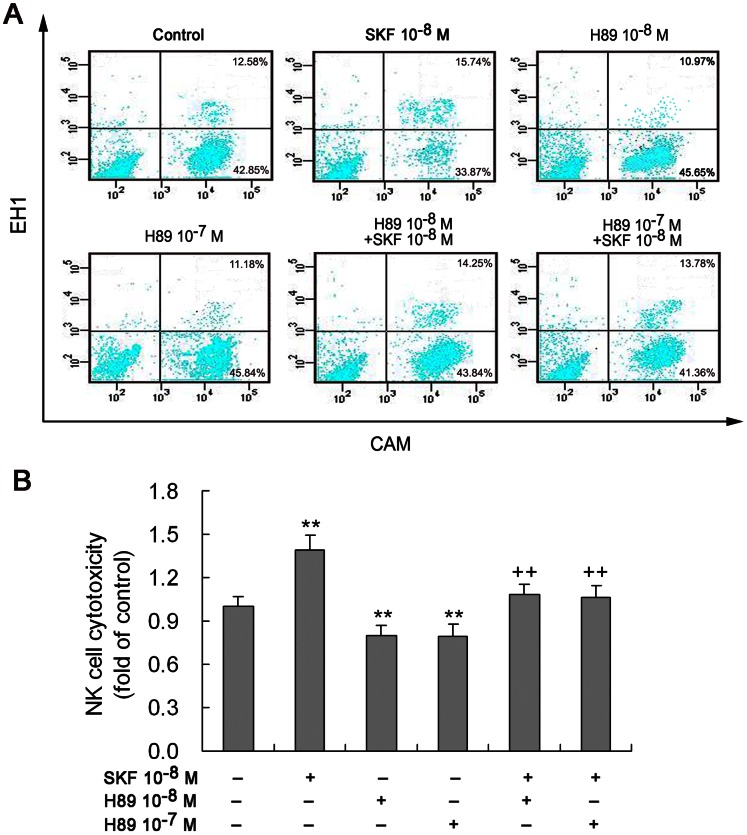
PKA inhibitor H89 prevents the SKF38393-dependent enhancement of NK cell cytotoxicity. NK cells that had been treated with PKA inhibitor H89 (10^−8^ or 10^−7^ M) or co-treated with H89 and D1-like receptor agonist SKF38393 (10^−8^ M) for 4 h were measured by flow cytometric analysis for the cytotoxicity against YAC-1 target cells. (A) A representative diagram showing cell percentage by the fluorescent dots in four plots of each treatment. The meaning of the cells in the four plots is the same as described in Fig. 2. (B) A statistical graph of eight independent experiments. **p<0.01, vs. control; ^++^p<0.01, vs. SKF (SKF38393).

To further demonstrate that the D1R/D5R-cAMP-CREB signaling pathway is involved in the modulation of NK cell function, we observed effect of PKA inhibitor H89 on NK cell cytotoxicity against YAC-1 target cells. In comparison with control lacking treatment, the exposure to H89 (10^−8^ or 10^−7^ M) attenuated NK cell cytotoxicity (Fig. 4). Moreover, the enhanced NK cell function by SKF38393 (10^−8^ M) was prevented by H89 (Fig. 4). These data confirm that cAMP-PKA signaling mediates D1R/D5R-stimulated NK cell function.

### D2-like Receptor Agonist Quinpirole Diminishes NK Cell Cytotoxicity and the Antagonist Haloperidol Blocks this Effect

**Figure 5:**
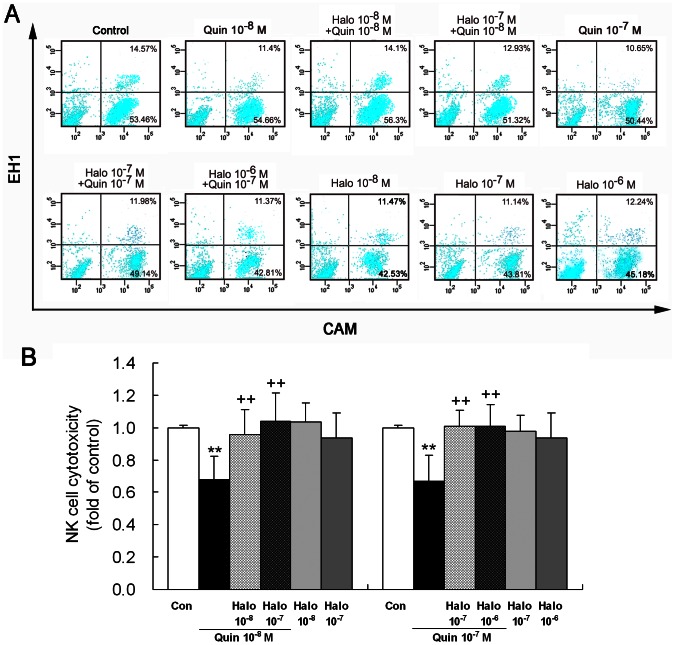
Activation of D2-like receptors attenuates NK cell cytotoxicity. NK cells that had been exposed to quinpirole (10^−8^ or 10^−7^ M), an agonist of D2-like receptors, or co-exposed to haloperidol (10^−8^, 10^−7^ or 10^−6^ M), an antagonist of D2-like receptors, and quinpirole for 4 h were examined by flow cytometric analysis for the cytotoxicity against YAC-1 target cells. (A) A representative diagram showing percentage of cells that were dead or intact NK cells and dead or living YAC-1 cells in the four plots of each treatment by the fluorescent dots. The explanation of the images is the same as shown in Fig. 2. (B) A statistical graph of eight independent experiments. **p<0.01, vs. control (Con); ^++^p<0.01, vs. Quin (quinpirole). Halo: haloperidol.

Treatment of NK cells with D2-like receptor agonist quinpirole (10^−8^ or 10^−7^ M) led to an attenuation of cytotoxicity of NK cells against YAC-1 target cells (Fig. 5). Co-treatment of NK cells with D2-like receptor antagonist haloperidol (10^−8^, 10^−7^ or 10^−6^ M) and agonist quinpirole (10^−8^ or 10^−7^ M) significantly augmented NK cell cytotoxicity with respect to the treatment with quinpirole alone, displaying a return to control level (Fig. 5). The haloperidol treatment alone at the three concentrations did not notably alter NK cell cytotoxicity (Fig. 5).

### Quinpirole Reduces D3R and D4R Expression, cAMP Content and CREB Phosphorylation in NK Cells, and these Effects are Blocked by Haloperidol

**Figure 6:**
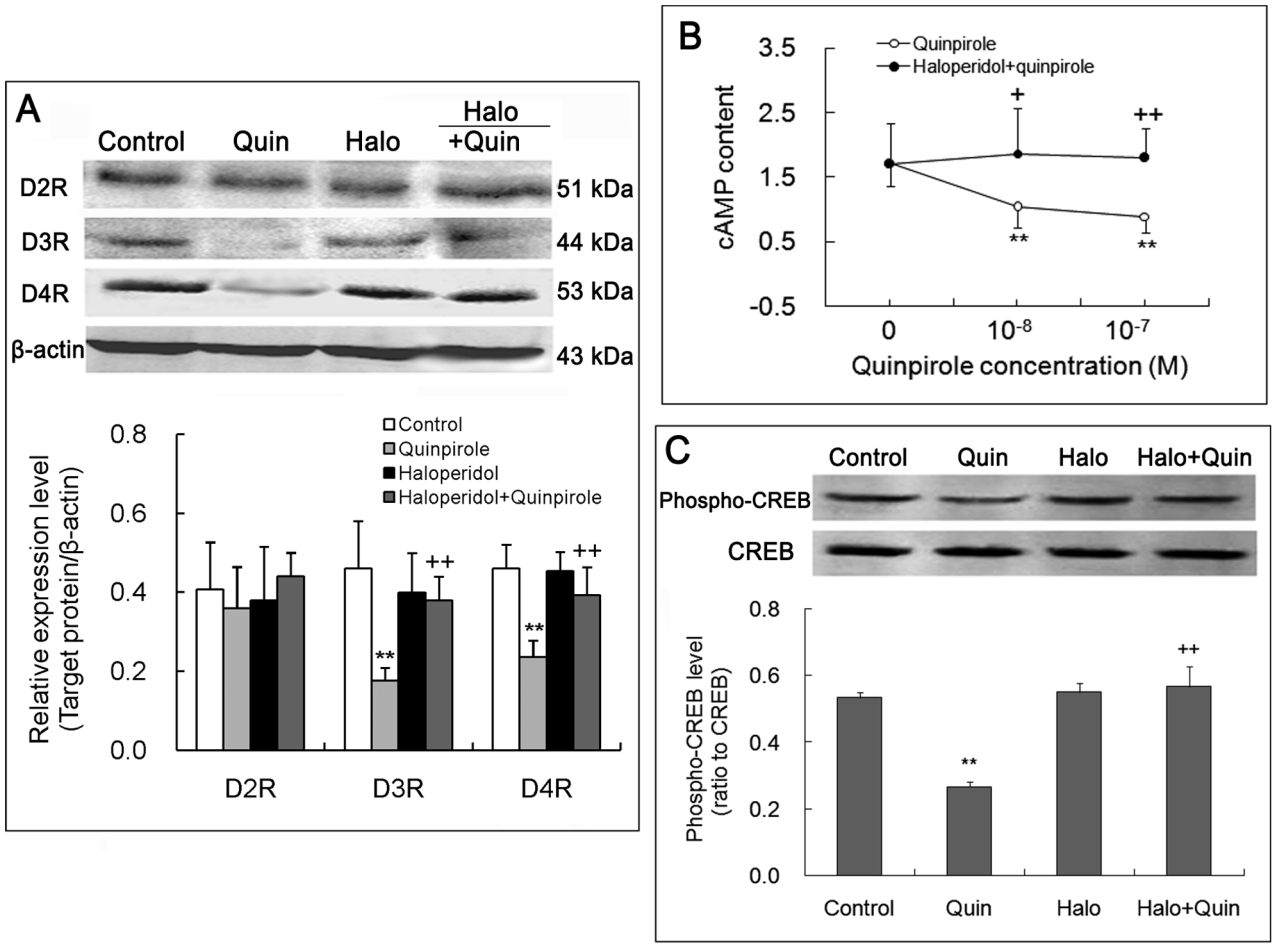
Activation of D2-like receptors reduces D3R and D4R expression, cAMP content, and phosphorylated CREB level in NK cells. NK cells were exposed to D2-like receptor agonist quinpirole (10^−8^ or 10^−7^ M) or co-exposed to antagonist haloperidol (10^−7^ M) and the agonist quinpirole for 4 h and then measured by Western blot and radioimmunoassay, respectively. (A) Representive electrophoretic bands and the statistic chart for three independent experiments. (B) Compilation of data for mean and standard deviation of eight respective experiments. (C) Representive electrophoretic bands and compilation of data obtained from three separate experiments. **p<0.01, vs. control; ^+^p<0.05, ^++^p<0.01, vs. quinpirole (Quin).

D2-like receptor agonist quinpirole (10^−8^ M) markedly down-regulated expression of D3R and D4R proteins but did not significantly decrease D2R expression in NK cells. Compared with the quinpirole exposure alone, the combined exposure to D2-like receptor antagonist haloperidol (10^−7^ M) and agonist quinpirole (10^−8^ M) up-regulated the expression of D3R and D4R proteins but did not significantly alter D2R expression (Fig. 6A), indicating a blockage of quinpirole-induced down-regulation of D3R and D4R expression by haloperidol. The treatment with haloperidol alone did not significantly affect the expression of the three subtypes of DA receptors (Fig. 6A).

Quinpirole (10^−8^ or 10^−7^ M) decreased cAMP content in NK cells, and this effect was abolished by haloperidol (10^−7^ M). Haloperidol exposure alone did also not influence cAMP level in NK cells (Fig. 6B).

Phosphorylated CREB level was significantly lower in quinpirole-treated NK cells than in control cells (Fig. 6C). The co-treatment with haloperidol (10^−7^ M) and quinpirole (10^−8^ M) caused the quinpirole-dependent decrease in CREB phosphorylation to return to control level (Fig. 6C). Haloperidol treatment alone did not alter CREB phosphorylation level.

### AC Activator Forskolin Enhances NK Cell Cytotoxicity and Counteracts the Quinpirole-dependent Suppression of NK Cytotoxicity

**Figure 7:**
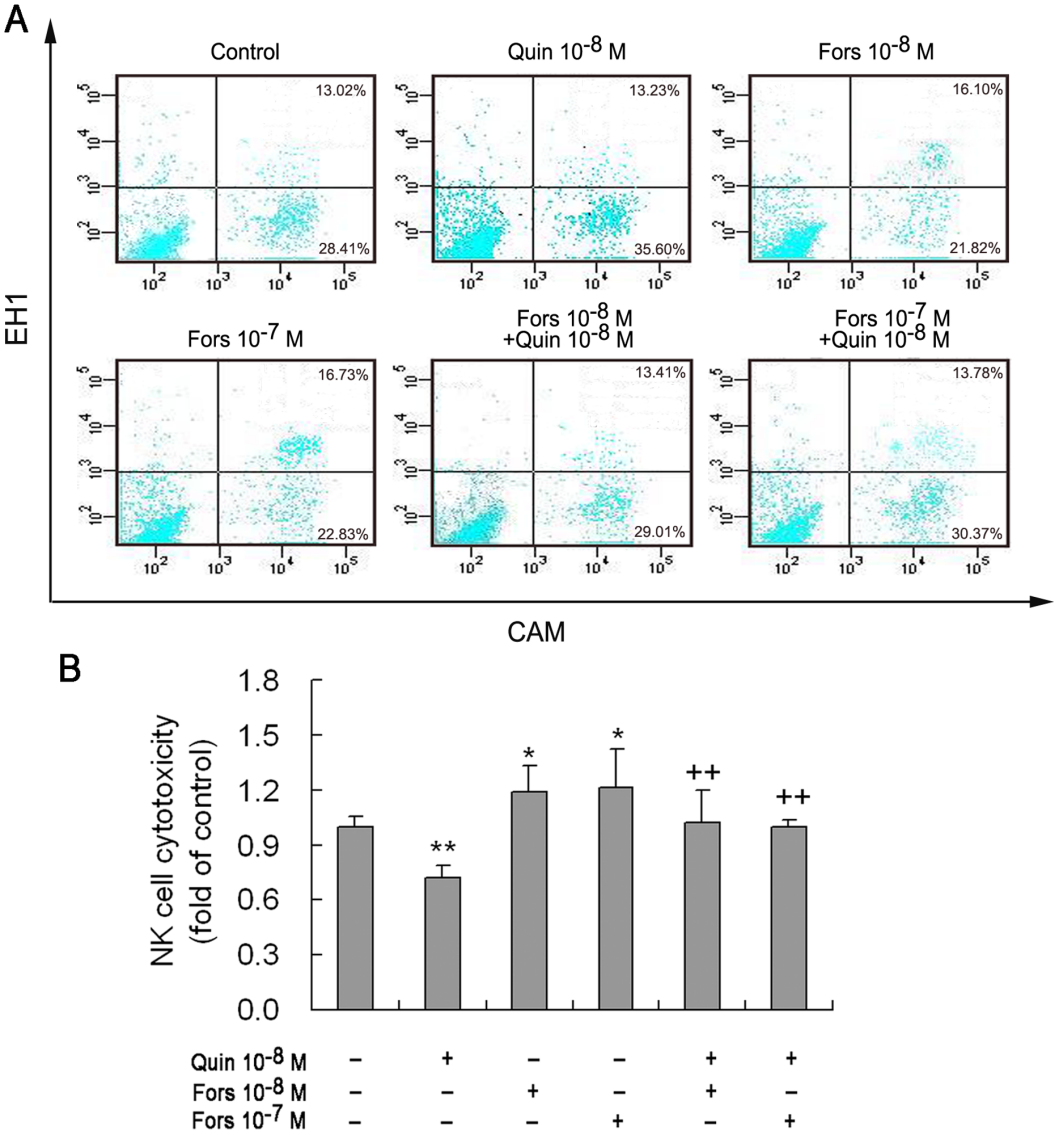
AC activator forskolin counteracts the quinpirole-dependent suppression of NK cell cytotoxicity. NK cells were treated with AC activator forskolin (10^−8^ or 10^−7^ M) or with forskolin plus quinpirole (10^−8^ M) for 4 h. (A) A representative diagram of fluorescent dot images reflecting cytotoxicity of NK cells against YAC-1 by flow cytometry, which was designed similarly to that in Fig. 2. (B) Statistics of eight independent experiments. *p<0.05, **p<0.01, vs. control; ^++^p<0.01, vs. Quin (quinpirole). Fors: forskolin.

Treatment of NK cells with forskolin, an activator of AC, augmented the cytotoxicity (Fig. 7). Combined treatment with forskolin and quinpirole restored the quinpirole-dependent inhibition of NK cytotoxicity almost to the control level (Fig. 7), showing a counteracting effect of forskolin on quinpirole. This demonstrates that the D2-like receptor-induced inhibition of NK cell cytotoxicity is implemented by suppression of cAMP-PKA signaling.

## Discussion

The five DA receptor subtypes, D1R-D5R, were expressed by NK cells in this study. It extends the evidence showing that D1R-D5R are expressed in murine NK cells. The existence of DA receptors on NK cells provides a substantial basis for the modulation of NK cell function by DA. Stimulation of D1-like receptors by the agonist SKF38393 enhanced NK cell cytotoxicity, whereas activation of D2-like receptors by the agonist quinpirole suppressed NK cells. These results suggest that the two families of DA receptors have opposite roles in mediating DA modulation of NK cells. This also explains a complicated effect of DA on immune cells. In our previous work, we have shown that D2-like receptors attenuate T cell proliferation and T helper 1 (Th1) cytokine production but augment Th2 cytokine production, while D1-like receptors only reduce Th1 cytokine production, suggesting that DA receptor subtypes play different roles in adjusting T lymphocytes [1]. Other reports support the suggestion. Activation of D1-like receptors attenuates cytotoxic effector function of cytotoxic T lymphocytes [14], while stimulation of D3 receptors facilitates differentiation of naïve CD8^+^ T cells towards cytotoxic T lymphocytes [17] and also enhances chemotaxis and adhesion of these cells [22], [23]. In addition to T lymphocytes, dentritic cells (DC) are modulated by DA receptor subtypes. D1-like receptor antagonists promote DC-dependent inhibition of pathogenic Th17-effector phenotype and favor Th1 differentiation, but antagonism of D2-like receptors in DCs increases their capacity to promote differentiation toward Th17 cells and reduce Th1 polarization [24]. Our present results showing that activation of D1-like receptors enhances NK cell cytotoxicity but activation of D2-like receptors suppresses NK cells provide more evidence for the distinct effects of DA on lymphocytes by the different DA receptor subtypes. Thus, due to the plentiful receptor subtypes, the limited neurotransmitters and hormones in the body can exert extensive and various regulatory effects. The mechanisms underlying the different effects caused by the different receptor subtypes are related to distinct signaling pathways activated by the receptors.

In general, D1-like receptors are positive regulator of intracellular cAMP levels by activating the enzyme AC [25], [26]. The cAMP induces PKA activation, and in turn the activated PKA phosphorylates cytoplasmic and nuclear proteins and regulates ion channel function and gene expression [18], [26], [27]. In contrast, D2-like receptors are negatively coupled to this cAMP/PKA-dependent signaling [8], [28]. In the current study, activation of D1-like receptors on NK cells by the agonist SKF38393 led to an up-regulation of D1R and D5R expression, an increase in cAMP content, and an elevation of phosphorylated CREB. These data confirm that D1-like receptors activate cAMP-CREB signaling pathway in NK cells. Importantly, the PKA inhibitor H89 attenuated NK cell cytotoxicity and also prevented the enhancement of NK cell cytotoxicity induced by the D1-like receptor agonist SKF38393. The findings further demonstrate that cAMP/PKA signaling mediates D1-like receptor-stimulated NK cell function. On the contrary, activation of D2-like receptors by the agonist quinpirole resulted in a down-regulation of D3R and D4R expression, a decrease in cAMP content, and a reduction of CREB phosphorylation level in NK cells. This verifies that D2-like receptors on NK cells, like those on other types of cells [29]–[32], are negatively coupled to cAMP-PKA-CREB signaling pathway. Notably, forskolin, an activator of AC that increases intracellular level of cAMP, enhanced NK cell cytotoxicity and also counteracted the quinpirole-dependent inhibition of NK cells. These results suggest that cAMP/PKA signaling suppression caused by D2-like receptor activation mediates the attenuation of NK cell function. Accordingly, the findings showing that the activation of cAMP-PKA-CREB signaling pathway facilitated NK cell cytotoxicity and suppression of this pathway impaired NK cells propose that cAMP-PKA-CREB signaling is actively involved in NK cell modulation by DA. On the other hand, these facts explain that although different DA receptor subtypes employ the same cAMP-PKA-CREB signaling pathway in NK cells, DA can exert opposite effects on the NK cells via activating different receptor subtypes that are positively or negatively coupled to the cAMP-PKA-CREB pathway. This phenomenon also occurs in T lymphocytes. Therefore, by stimulating DA receptors, DA can regulate T cell function either negatively or positively [19].

In addition to cAMP-PKA-CREB, other signaling pathways are also involved in modulation of T lymphocytes by DA. PKA and cAMP inhibit extracellular signal-regulated kinase (ERK) phosphorylation [33] and c-jun N-terminal kinase (JNK) activation [34], activate C-terminal Src kinase [35], and block nuclear factor-κB (NF-κB) activation [36], [37]. All of these intracellular biochemical events induce a marked impairment on T cell activation with inhibition of T cell proliferation and cytokine production [38]. Since activation of PKA and CREB can inhibit translocation of NF-κB [39], DA-induced CREB activation also results in diminished NF-κB-dependent transcription and hence in an impairment of inflammatory response [40]. However, stimulation of D2-like receptors activates NF-κB [41], suggesting that this DA receptor subpopulation has a facilitating effect on inflammatory response. These findings demonstrate that DA receptor subtypes employ various downstream signaling pathways to perform complicated modulation of lymphocytes. Further functional significance of these signaling pathways in DA modulation of lymphocytes particularly NK cells still needs to be explored.

In summary, NK cells expressed all the five subtypes of DA receptors, including D1R and D5R (D1-like receptor family) and D2R, D3R and D4R (D2-like receptor family). Activation of D1-like receptors by the agonist SKF38393 significantly enhanced cytotoxicity of NK cells against YAC-1 lymphoma cells, while activation of D2-like receptors by the agonist quinpirole suppressed the NK cells. Moreover, the activation of D1-like receptors stimulated cAMP-PKA-CREB signaling pathway, whereas the activation of D2-like receptors inhibited this signaling pathway. Significantly, the stimulation of the cAMP-PKA-CREB signaling pathway abolished the D2-like receptor-dependent suppression of NK cells, while the inhibition of this signaling pathway prevented the D1-like receptor-induced enhancement of NK cells. These findings suggest that D1-like and D2-like receptors have opposite roles in modulation of NK cell cytotoxicity and that the opposite effects are attributed to their positive or negative links to cAMP-PKA-CREB signaling pathway, respectively.
